# A Temporal Adaptive Access Mechanism for Data Fusion in an IoT Environment

**DOI:** 10.3390/s18124205

**Published:** 2018-11-30

**Authors:** Jiuyun Xu, Shuang Liu, Xiaoxuan Lu, Li Li, Hongliang Liang, Qiang Duan, Runjie Liu

**Affiliations:** 1 College of Computer & Communication Engineering, China University of Petroleum (East China), Tsingdao 266580, China; shuangliu820@gmail.com (S.L.); luxx01@163.com (X.L.); lily2008@upc.edu.cn (L.L.); 2 Department of Informatics, Beijing University of Posts and Telecommunications, Beijing 100876, China; hliang@bupt.edu.cn; 3 Information Sciences and Technology Department, Pennsylvania State University, Abington, PA 19001, USA; qxd2@psu.edu; 4 Institute of Smart City, Zhengzhou University, Zhengzhou 450001, China; ierjliu@zzu.edu.cn

**Keywords:** Internet of Things, data fusion, real/non-real time, distributed scheduling algorithm

## Abstract

Data fusion in the Internet of Things (IoT) environment demands collecting and processing a wide variety of data with mixed time characteristics, both real-time and non-real-time data. Most of the previous research on data fusion was about the data processing aspect; however, successful data transmission is a prerequisite for high-performance data fusion in IoT. On the other hand, research on data transmissions in IoT mainly focuses on networking without sufficiently considering the special requirements of the upper-layer applications, such as the data fusion process, that are consuming the transmitted data. In this paper, we tackle the problem of data transmission for data fusion in an IoT environment by proposing a distributed scheduling mechanism VD-CSMA in wireless sensor networks, which considers the values for data fusion, as well as the delay constraints of packets when determining their priority levels for transmission. Simulation results have shown that VD-CSMA may enhance both throughput and delay performance of data transmission as compared to the typical scheduling schemes used for data fusion in IoT.

## 1. Introduction

With the rapid development of the Internet of Things (IoT) [1,2], data fusion technologies have been widely employed to process the huge amount of data generated in an IoT environment; thus becoming a significant component for supporting a wide variety of IoT-based applications [3,4]. Data fusion is a multilevel multifaceted process that handles the automatic detection, association, correlation, estimation, and combination of data and information from multiple sources [5]. The advantages of data fusion include expanding time and space coverage of data sources, improving identification of the system’s viability, enhancing target recognition rate, increasing confidence, reducing ambiguity, and being able to utilize sensor network resources fully. Data fusion aggregates the data collected from multiple sources for information analysis to obtain more precise estimation, accurate prediction, and optimal decision-making for enhancing IoT service performance. Therefore, data fusion has become an active research topic that has attracted interest from both academia and industry.

In an IoT environment, the huge amount of data processed by data fusion is typically collected by a large number of sensors. Therefore, wireless sensor networks [6] become an important ingredient for an IoT data fusion system. A wireless sensor network typically comprises a set of sensor nodes with sensing, computing, and communication capabilities. Sensor nodes collect multiple types of data about the surrounding environment and send the data to a fusion center, which processes the data and performs comprehensive analysis [7]. A primary reason that makes wireless sensor networks attractive to data fusion in IoT is their ability to monitor physical environments through the ad hoc deployment of numerous tiny, intelligent, and wireless sensor nodes; thus supporting data fusion in a wide range of scenarios, such as in military applications [8,9], environmental monitoring [6], health care [10,11], intelligent homes [12], and security protection [13].

Data fusion in an IoT environment comprises two closely-related aspects: transmitting collected data to a fusion center and processing data at the fusion center to obtain useful information for supporting applications. Previous research on data fusion mainly focused on the latter aspect with works on theory and methods for data estimation, statistical methods, information theory, artificial intelligence, etc. [14]. However, successful data transmission from sensors to a fusion center, typically through a wireless sensor network, is a prerequisite for high-performance data fusion in IoT. Data transmissions in wireless sensor networks for supporting data fusion have some special requirements. Although the wireless sensor network has been extensively studied in the field of networking research, most of the existing works focused on networking mechanisms without sufficiently considering the special requirements of the upper-layer applications, such as the data fusion process, that are consuming the transmitted data. Therefore, data transmission in wireless sensor networks for supporting data fusion in an IoT environment is still an open issue that deserves more thorough investigation, which is the subject that we will study in this article.

Data fusion in IoT demands various types of data with diverse time characteristics (real time and non-real time) to be transmitted from sensors to a fusion center. Real-time data must be delivered to the fusion center with a strict delay constraint. Real-time data that failed to reach the fusion center may cause a serious decline in data fusion performance. In a situation where packet delay and discard are inevitable due to heavy traffic load, it becomes essential to guarantee transmission of the packets that are most valuable from a data fusion perspective in order to ensure data fusion performance. Therefore, how to evaluate the value of a packet in terms of its importance to data fusion becomes an important topic to investigate.

We observed that it is the changes in data carried by a packet that provide new information to data fusion; therefore, a packet containing data with more frequent changes would be more valuable from a data fusion perspective. The frequency of data changing is referred to as the data correlation of a packet in this paper and used as a measure of the value of a packet. A packet with a small correlation (the data bits contained in the packet have less relevance) is more valuable for data fusion than a packet with large correlation (the data bits in this packet are highly relevant to each other). In order to guarantee the performance of data fusion, it is necessary to prioritize the transmission of data packets that are more valuable for data fusion. How to allocate wireless network resources according to the different characteristics of various data to meet their different QoS requirements is one of the problems that radio resource allocation must consider. In particular, when real-time and non-real-time data with different QoS requirements coexist in a wireless sensor network, resource allocation issues with QoS considerations are particularly important.

In this paper, we tackle the problem of data transmission for data fusion in an IoT environment by proposing a scheduling mechanism in a wireless sensor network, which considers the values for data fusion, as well as the delay constraints of packets when determining their priority levels for transmission. Specifically, we make the following contributions in this paper.

- We investigate the data transmission problem in wireless sensor networks from the perspective of data fusion and advocate a new method of data transmission for enhancing data fusion performance; thus integrating the two aspects—data transmission and data process—of data fusion in an IoT environment.
- We propose a dynamic priority assignment scheme that determines the priority level for each packet based on multiple factors, including the value of the packet for data fusion, which is measured by the data correlation of the packet, as well as the urgency of the packet with respect to its transmission deadline. This scheme assigns higher priority levels to packets that contain more valuable data for data fusion and closer to their deadlines; thus allowing such packets to obtain better chances for transmission in a distributed scheduling system.
- We develop a time-adaptive scheduling algorithm VD-CSMA based on the dynamic priority assignment, which can enhance throughput and delay performance for real-time data transmission and also ensure transmission of more valuable packets for data fusion. Extensive simulation evaluations have been conducted, and the obtained results verified that the proposed algorithm achieves good throughput and delay performance under both light and heavy traffic loads.

The rest of the paper is organized as follows. Section 2 reviews related works. Section 3 presents the network model we use in this work. The dynamic priority mechanism is presented in Section 4, while the distributed scheduling algorithm VD-CSMA is developed in Section 5. Simulation results are presented and analyzed in Section 6. We draw conclusions in Section 7.

## 2. Related Work

Extensive research work has been done on multi-sensor data fusion; for example, a theoretical approach to data fusion [15], a fusion model [16,17], and data processing [18]. Most of the work focused on data acquisition and processing in data fusion. Data communications from sensors to the data fusion center are also a key component of the data fusion process and have a significant impact on data fusion performance.

Considering communications in a resource-constrained wireless sensor network, efficient resource allocation plays an important role in achieving high performance networking for supporting data fusion; thus forming an important research direction [19]. It is well known that the Maximum Weighting Scheduling (MWS) algorithm based on queue length [20] can stabilize all network queues within the network capacity area. However, MWS it is not suitable for large-scale networks due to its complexity, which requires a centralized implementation. Some suboptimal algorithms with lower complexity have been proposed to address the scalability issue, among which is the Greedy Maximum Scheduling algorithm (GMS) [19,21], which has been widely used in wireless networks. However, performance analysis has shown that GMS can only achieve a small part of the capacity area. The work reported in [22] indicates that GMS may achieve optimal throughput in networks that satisfy the conditions of local pool, but not in networks with a general topology. In [23], the authors studied the scheduling efficiency of distributed scheduling strategies in wireless networks based on the concept of greedy scheduling and found that only a small number of throughput regions can be implemented after ignoring collisions.

Compared to distributed scheduling algorithms, centralized scheduling schemes often result in better performance at the expense of higher complexity by requiring central authorities to allocate network resources. In a large-scale sensor network, the centralized scheduling scheme is extremely difficult to implement. Therefore, an efficient distributed scheduling algorithm can still be attractive even if it can only obtain suboptimal performance compared to centralized scheduling schemes.

Random access protocols play a key role in wireless sensor networks due to the low cost and easy deployment [24]. Carrier Sense Multiple Access (CSMA) is a type of distributed scheduling scheme for random access to shared data transmission channels. With CSMA, each sensor will detect whether the channel is busy before transmitting a packet. When a sensor detects a busy channel, it will wait for a random backoff time. CSMA is widely used in practice because of its easy implementation in a distributed fashion. The distributed adaptive CSMA scheduling algorithm proposed in [25] may achieve maximum throughput. It was shown in [26] that CSMA random access can achieve the maximum possible throughput in a wireless ad hoc network. The authors of [27] introduced activity probability in their performance analysis model and developed a contention window control scheme that achieved the ideal contention status in an average sense. The schemes proposed in [27,28] achieved considerable throughput improvement. However, these algorithms achieved high throughput by sacrificing their delay performance, especially under heavy traffic loads.

On the other hand, some research has been done for improving CSMA delay performance. For example, the delayed CSMA scheme proposed in [29] utilized past state information observed by a local link to construct a high-order Markov chain for the evolution of the feasible link schedules. The algorithm developed in [30] applied quadratic Lyapunov functions in conjunction with the Lindley equation and Azuma’s inequality for obtaining an exponential decaying property in certain queueing dynamics. Although some simple heuristics can produce better delay performance, they usually only reach a fraction of the capacity area. For the problem of communication in data fusion, the DGCSMA algorithm proposed in [31] took into account the time constraints and the historical transmission statistics of the sensor. This algorithm achieved better delay performance, but assumed the availability of infinite buffer space.

The aforementioned research either focused on fusion of the data received by the fusion center without considering the impact of data transmission or attempted to enhance throughput and/or delay performance for data transmissions in wireless sensor networks without consideration of the special features of data fusion.

## 3. Network Model

**Figure 1:**
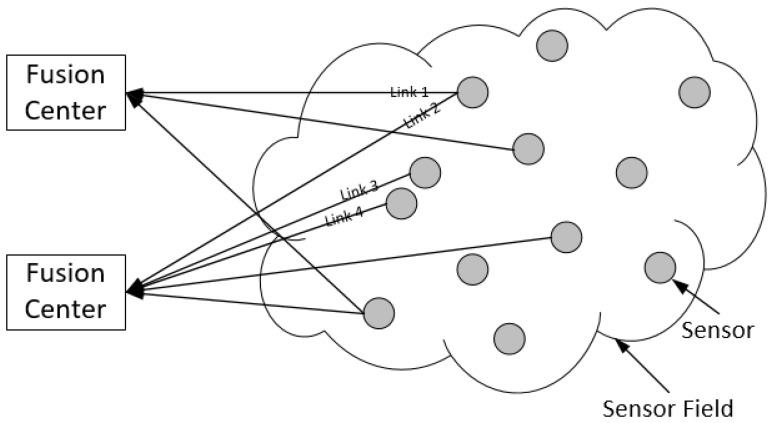
Wireless sensor network architecture for data fusion in IoT.

In this paper, we consider an IEEE 802.11-based wireless sensor network as the data transmission infrastructure for data fusion in an IoT environment. Figure 1 depicts the architecture for such a networking scenario for data fusion. Each node in the network can send and receive data packets and can forward received packets to a nearby node with less traffic to avoid congestion. In this paper, we focus our study on a single-hop networking case—the last hop from sensor nodes to the fusion center—which provides asynchronous data transmission from a set of sensor nodes to a fusion center. In addition, we assume that some error-control mechanism has been applied either in the physical layer or in a cross-layer fashion; therefore, data packets are transmitted through ideal channels from a scheduling perspective.

**Figure 2:**
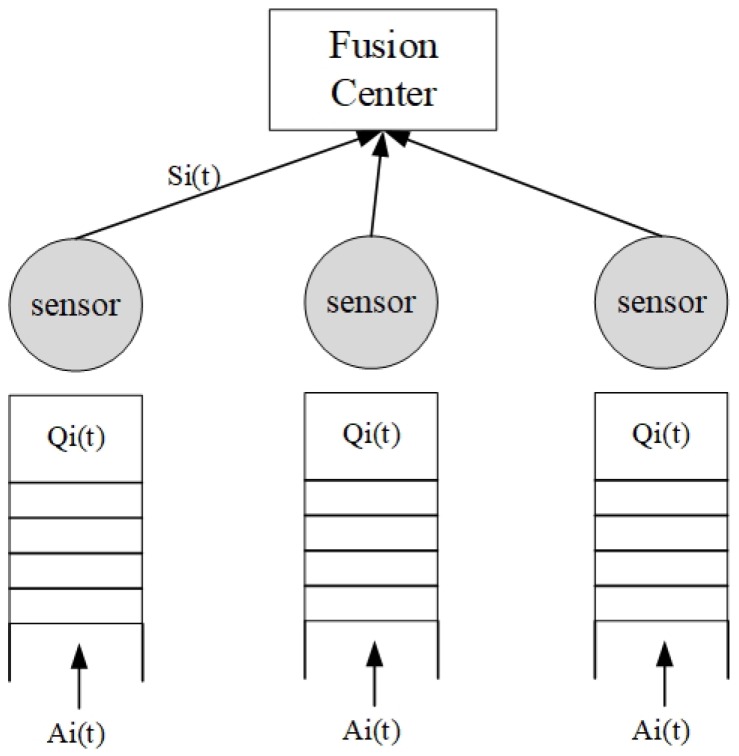
Scheduling process of the decision packets between the sensor and the fusion center.

The network architecture is modeled as an undirected graph G=(ν,ε), where ν is the set of sensors and the fusion center and ε is the set of links. ε contains real-time links and non-real-time links, which are respectively dedicated to transmitting real-time and non-real-time packets. $L_{i}$ is the $i^{\mathrm{th}}$ link, i∈{1,⋯,∥ε∥}, from a sensor to the fusion center. Each link has a queue with an exogenous arrival process through which new data arrive in the form of unit-sized packets. When a packet is generated by a sensor, it enters the queue of the corresponding link and waits for transmission. The $j^{\mathrm{th}}$ packet in the $i^{\mathrm{th}}$ link is expressed as $p_{ij}$. Figure 2 shows the scheduling process that determines the sequence in which packets are transmitted from a set of sensor nodes to a fusion center.

At the beginning of each slot, the backlog of the queue for $L_{i}$ is represented by $Q_{i}\left(t\right)$, $Q_{i}\left(t\right)\geq 0$, $Q_{i}\left(0\right)=0$. We assume that each queue has a finite buffer limit Lt. When a new packet arrives at the queue, the queue length will increase if $Q_{i}\left(t\right)<Lt$; otherwise, the newly-arrived packet will be discarded. The number of packets discarded due to the buffer limit by slot *t* is recorded as $O_{i}\left(t\right)$ for link $L_{i}$.

The number of packet that arrives at $L_{i}$ in time slot *t*, denoted as $A_{i}\left(t\right)$, has an independent and identical Bernoulli distribution across all links. The service vector is represented by $S_{i}\left(t\right)$, and $L_{i}$ is served when $S_{i}\left(t\right)$ is one. The number of packet dropped from link $L_{i}$ in slot tis defined as $D_{i}\left(t\right)$. A delay threshold is assigned to each real-time packet by its source sensor. Real-time packets that cannot be successfully transmitted to the fusion center by their delay thresholds will be discarded. For non-real-time data, the delay threshold is assumed to be the upper limit of the delay threshold, and the non-real time data successfully received by the fusion center beyond the delay threshold are not discarded. Such a delay threshold setting is important for real-time QoS guarantees since real-time data that cannot be delivered to the fusion center on time will become useless for data fusion. The delay threshold setting for non-real-time data is to allow them to participate in channel competition to ensure fair access to the shared link capacity.

Since an ideal channel is assumed for data transmissions in the network, there are only two reasons for packets to be discarded: (i) a newly-arriving packet to a queue is discarded if the current queue length reaches the buffer limit; and (ii) a real-time packet is dropped if it cannot be successfully transmitted to the fusion center by its deadline. At the end of each slot, link *i* discards all real-time packets that exceed their delay threshold. The queue length for queue i∈{1,⋯,∥ε∥} is given by:

(1) $$Q_{i}\left(t+1\right)=max\left[Q_{i}\left(t\right)-S_{i}\left(t\right)-D_{i}\left(t\right),0\right]+\left(A_{i}\left(t\right)-O_{i}\left(t\right)\right)$$

In such a network, a group of sensors shares a finite set of channels for transmitting data to a fusion center. Therefore, the scheduling constraint here is that no two links that might interfere with each other can transmit data in the same time slot. For each link $L_{i}$, we use C(i) to denote the conflict set for the link; that is, the set of links such that if any one of them is active, then link $L_{i}$ cannot be active. The conflict set for a link includes:

- links that share a common node with this link (such as Link 1 and Link 2 in Figure 1). This models the node-exclusive constraint where two links sharing a common node cannot be active simultaneously;
- links that will cause interference to the link when transmitting (such as Link 3 and Link 4 in Figure 1). This models the radio interference constraint where two links that are close to each other cannot be active simultaneously.

Note that a feasible schedule satisfies $x_{i}+x_{j}\leq 1$, for all i∈Eandj∈C(i), where $x_{i}=1$ if link $L_{i}$ is selected by the scheduling scheme for data transmission and $x_{i}=0$ otherwise. Therefore, a solution to the following optimization problem gives a set of links that comprise the maximum number of links for packet transmissions without interference in a time slot, which is referred to as the maximum scheduling set.

$$\begin{array}{c} \max\;\;\sum_{i=\varepsilon }P_{i1}x_{i}\\ s.t.\;\left\{\begin{array}{c} x_{i}+x_{j}\leq 1\\ i\in E\;and\;j\in C(i). \end{array}\right. \end{array}$$

where $P_{i1}$ is the priority level of the packet with the highest priority in link *i*.

## 4. Dynamic Priority Assignment

Considering the diverse types of data collected by sensor nodes and processed by the fusion center in a general IoT environment, transmissions of packets containing different data will have different impacts on the performance of data fusion. It is critical to transmit real-time packets carrying time-critical data to the fusion center by certain deadlines; otherwise, the data become useless in the data fusion process. In addition, parts of the collected data represent significant changes in status of the monitored objects, which makes the packets carrying such data more important from a data fusion perspective. Therefore, the scheduling mechanism for data transmissions in wireless sensor networks should take into account the relative importance of packets in order to meet the requirement for data fusion. Toward this direction, we propose a dynamic priority assignment scheme in this section, which evaluates the “value” of a packet that reflects its significance to data fusion; thus assigning higher priority to packets carrying more valuable data to the data fusion process.

Without loss of generality for our study, we assume that the sensor will make a local decision by comparing each sensor datum with a specified threshold in this paper. A sensor sends a local decision packet with local decision values of zero or one instead of the specific detection result to the fusion center. If the detection result is greater than the threshold, the decision is one; otherwise, the decision is zero. The decision values are packaged into fixed size packets. The received signal at sensor *k* can be expressed as:

(2) $$s_{k}\left(t\right)=a_{k}\left(t\right)+n_{k}^{s}\left(t\right)$$

where $a_{k}\left(t\right)$ is the sensor detection signal and $n_{k}^{s}\left(t\right)$ is Gaussian noise.

Let $x_{k}\left(t\right)$ be the local decision made by the sensor, then:

(3) $$x_{k}\left(t\right)=I$$

$$I=\left\{\begin{array}{c} 1\;if\;s_{k}\left(t\right)\geq T_{H}^{k}\\ 0\;if\;s_{k}\left(t\right)<T_{H}^{k} \end{array}\right.$$

where *I* is the indicator function. Equation (3) specifies how a sensor compares the specific detection result with the pre-specified threshold. When $s_{k}\left(t\right)$ is greater than or equal to the pre-specified threshold $T_{H}^{k}$, the local decision is one, and when $s_{k}\left(t\right)$ is less than $T_{H}^{k}$, the local decision is zero.

The series of binary decision bits in each packet is denoted as $Pd_{ij}=\left\{b_{1},b_{2},b_{3},\cdots ,b_{n}\right\}$, where $b_{n}$ is a decision value of zero or one within the packet, which can be determined following (3). Each packet consists of *n* binary decision values.

The decision-making relevance of a decision packet (a packet contains a series of decisions $Pd_{ij}$) is calculated as the eXclusive-OR of all the decision values within the packet and accumulates the exclusive-OR results to get $Xp_{ij}$. The correlation parameter $C_{ij}$ of a decision packet is determined as the ratio of $Xp_{ij}$ to the total number of XOR calculations needed for obtaining $Xp_{ij}$; that is,

(4) $$Xp_{ij}=b_{1}\wedge b_{2}\wedge b_{3}\ldots b_{n-1}\wedge b_{n}$$

(5) $$C_{ij}=\frac{Xp_{ij}}{n-1}\;$$

For some common applications such as environment detection and target tracking, a sudden change in decision values plays an important role in the decision-making of data fusion. Therefore, the value of each decision packet varies from the perspective of data fusion. In this paper, the parameter $C_{ij}$ of a packet is used as a measure of the value of the packet to data fusion. A smaller $C_{ij}$ value means less changes in the adjacent local decision data in a packet, which implies stronger correlation of the decision values within the packet; therefore, the packet is less valuable to the data fusion process. A larger $C_{ij}$ value means more frequent changes in adjacent local decision data in the packet and thus weaker correlation of the decision values within the packet, which makes the packet more valuable to data fusion.

Real-time data are time-bound and sensitive to delay; thus having a deadline threshold. Packets containing real-time data must be successfully transmitted to the fusion center by the deadline; otherwise, they will be discarded. The dynamic priority assignment scheme proposed in this paper determines the priority for a packet by weighting two parameters: the urgency of this packet measured by the remaining life-time of the packet and the correlation of the packet calculated following (5). In addition, the historical miss rate of the link is also taken into account for assigning priority for a packet.

In order to meet the performance requirements of data fusion, the transmission priority for a decision packet cannot be determined solely by either the deadline of the decision packet or the value of the decision packet because the most urgent packet is not necessarily the most valuable for data fusion. Transmitting the most valuable decision packet first will cause time-critical packets to be discarded due to deadline violation. Therefore, the real-time data scheduling algorithm follows the following principle: when the remaining lifetime of the real-time packet is long, the data relevance parameter of the packet plays a major role in dynamic priority assignment. For real-time packets close to their deadlines, the time urgency plays a major role in determining priority for the packet. That is, under the condition that the real-time data are time-constrained, packets with large correlation parameters are transmitted as much as possible. This ensures that a decision packet with a big value can be transmitted with a high priority.

There is no deadline for non-real-time packets to be discarded due to lifetime expiration. However, if the network is busy and there are too many decision packets, there is no way to transfer all data within the expected time. For the fusion center, in order to ensure the performance of data fusion, decision packets that are more valuable for data fusion need to be transmitted with higher priority levels. In this paper, the priority is defined for each packet in each link so that all packets on each link are sorted by priority before entering the channel. The purpose is to be more effective according to the priority of the packet to ensure the quality of transmission, which can greatly improve the channel utilization.

The priority for a real-time packet $p_{ij}$ in time slot *t*, denoted as $P_{ij}\left(t\right)$, is determined as:

(6) $$P_{ij}\left(t\right)=\delta {}^{\frac{T_{ij}\left(t\right)}{Td_{ij}}}+C_{ij}+\omega_{1}\left(M_{i}^{k}\left(t\right)+V_{i}^{k}\left(t\right)\right)$$

where $T_{ij}\left(t\right)$ and $Td_{ij}$ are respectively the remaining lifetime and expiration time of the packet and *t* is the current time. The smaller the ratio of $T_{ij}\left(t\right)$ to $Td_{ij}$ is, the shorter the remaining lifetime a packet has and thus the more urgent it is for the packet to be transmitted. The parameter δ takes its value from the interval (0,1) to reflect the urgency aspect in priority assignment in order to give a packet with a smaller $T_{ij}\left(t\right)$ to $Td_{ij}$ ratio higher priority to gain a better chance to be scheduled for transmission.

The priority for a non-real-time packet is defined as follows:

(7) $$P_{ij}\left(t\right)=C_{ij}+\omega_{2}\left(M_{i}^{k}\left(t\right)+V_{i}^{k}\left(t\right)\right)$$

$T_{ij}\left(t\right)$ is the remaining lifetime of the packet in slot tand can be calculated as follows:

(8) $$T_{ij}\left(t\right)=Ta_{ij}+Td_{ij}-t$$

where $Ta_{ij}$ is the generation time of the packet.

$M_{i}^{k}\left(t\right)$ is the historical missing ratio for link $L_{i}$, which is defined as the ratio of the total number of packets that have missed their delay deadlines in the last *K* HOLpackets [31]; that is,

(9) $$M_{i}^{k}\left(t\right)=\frac{\sum_{j=1}^{K}I_{ij}\left(w_{ij}\left(t\right)\geq Td_{ij}\right)}{K}$$

$$I=\left\{\begin{array}{c} 1\;if\;w_{ij}\left(t\right)\geq Td_{ij}\\ 0\;if\;w_{ij}\left(t\right)<Td_{ij} \end{array}\right.$$

where $I_{ij}\left(\cdot \right)$, j∈K, is the indicator function, and $w_{ij}\left(t\right)$ is the waiting time of $p_{ij}$ since it is generated by a sensor.

The other factor we considered for dynamic priority assignment in (6) and (7) is $V_{ij}^{k}$, which represents the historical value missing rate, which is defined as the ratio of the sum of correlation values of all the missing packets in the last KHOL packets to the total correlation values of all the K packets; that is,

(10) $$V_{i}^{k}\left(t\right)=\sum_{j=1}^{K}\frac{I_{ij}\left(w_{ij}\left(t\right)\geq Td_{ij}\right)C_{ij}}{\sum_{j=1}^{K}KC_{ij}}$$

$$I=\left\{\begin{array}{c} 1\;if\;w_{ij}\left(t\right)\geq Td_{ij}\\ 0\;if\;w_{ij}\left(t\right)<Td_{ij} \end{array}\right.$$

The deadline for real-time decision packets in this paper is determined by the sensor, and the deadline for non-real-time packets is set to a predetermined value. A real-time packet is discarded when its waiting time exceeds its deadline and it has not been successfully received by the fusing center. When a non-real-time packet’s waiting time exceeds the specified deadline, its remaining lifetime $T_{ij}\left(t\right)$ will be set to zero, and it will be counted as a missing packet for calculating $M_{i}^{k}\left(t\right)$ and $V_{i}^{k}\left(t\right)$ using (9) and (10). However, the packet will not be discarded. Using $T_{ij}\left(t\right)$ for non-real-time packets is to avoid the link starvation issue in the proposed scheduling scheme.

## 5. Distributed Scheduling Algorithm

The scheduling algorithm developed in this section is based on the wireless network standard IEEE 802.11 CSMA/CA (Carrier Sense Multiple Access/Collision Avoidance). The developed algorithm explicitly handles packet collisions. By utilizing the dynamic priority assignment scheme proposed in the previous section, this scheduling algorithm favors packets that have strict delay constraints and carries more valuable data; thus, it may enhance packet transmission performance for meeting data fusion requirements.

In the scheduling system, the decision packets generated at sensor nodes can be thought of as consumers who compete for accessing the transmission link capacities as the shared resources. We divide each time slot into a control sub-slot and a transmission sub-slot (later, we will further divide the control sub-slot into control mini-slots). The purpose of the control sub-slot is to generate a collision-free transmission schedule used for data transmission in the transmission sub-slot. To achieve this, the scheduling scheme selects a set of links that do not conflict with each other, which is called the scheduling set in this time slot. Each control sub-slot is divided into *B* frames, and each frame is further divided into *W* mini-slots. $P_{i1}$ is divided into different intervals and mapped to different frames. Each link $L_{i}$ will randomly select the backoff time $w_{i}$ in the specified mapped frame according to $P_{i1}$. $w_{i}$ is defined as [31]:

(11) $$w_{i}=f\left(w\right)+NW$$

where *w* follows a discrete uniform distribution over [0,W−1] and N∈{0,1,⋯,B−1} is determined by the pre-specified mapping based on $P_{i1}$.

Algorithm 1 is as follows: 

**Algorithm 1** VD-CSMA.**Input:** The set of decision packets in link i;**Output:** The maximum scheduling set;
1:At the beginning of the control sub-time slot;2:**for** each packet in link *i* **do**3: According to the value of their own $P_{ij}$ arranged in the queue corresponding position;4: The final link *i* in the order of the packet is arranged according to $P_{ij}$ in descending order;5:
**end** **for**
6:The link *i* randomly selects the backoff time $w_{i}$ in the designated frame according to the priority of the first decision packet of the link (that is, the decision packet with the highest priority of the link) and waits for $w_{i}$ control sub-slots.7:**if** link *i* hears an INTENT message from a link in C(i) before the ${\left(w_{i}+1\right)}^{\mathrm{th}}$ control mini-slot, **then**8: Link *i* will not be included in the scheduling set and will not send (broadcast) an INTENT message in this slot;9:
**end** **if**
10:**if** link *i* does not hear an INTENT message from any link in C(i) before the ${\left(w_{i}+1\right)}^{\mathrm{th}}$ control mini-slot, **then**
11: Link *i* will send (broadcast) an INTENT message to all links at the beginning of the ${\left(w_{i}+1\right)}^{\mathrm{th}}$ control mini-slot.12: **while** there is a collision (i.e., if there is another link in C(i), transmitting an INTENT message in the same mini-slot) **do**13:  Link *i* will not be included in the scheduling set.14: **end** **while**15: **while** there is no collision **do**16:  Link *i* will be included in the scheduling set.17: **end** **while**18:
**end** **if**
19:**if** link *i* is picked in the scheduling set **then**20: Send the first decision packet of the link *i* to the fusion center in the transmission sub-slot.21: This scheduling set is the maximum scheduling set.22:
**end** **if**



The output of Algorithm 1 is the maximum scheduling set, that is the set of links with the highest priority and no interference that are found in the time slot *t*. In Algorithm 1, Lines 1–5 order packets in link *i* according to their priority levels. Lines 6–9 handle the case where link *i* hears an INTENT message from a link in C(i) before the ${\left(w_{i}+1\right)}^{\mathrm{th}}$ control mini-slot. Lines 10–18 are for the case where link *i* does not hear an INTENT message from any link in C(i) before the ${\left(w_{i}+1\right)}^{\mathrm{th}}$ control mini-slot. Lines 11–14 describe a procedure for responding collision occurrence, and Lines 15–18 give the process if no collision occurs. Lines 19–22 form the maximum schedule set of links that can simultaneously transmit packets to the fusion center in this time slot. The proposed algorithm is a distributed algorithm with complexity of O(n). This algorithm does not require a centralized controller; thus, it is easy to implement.

## 6. Performance Evaluation

### 6.1. Simulation Setting

**Figure 3:**
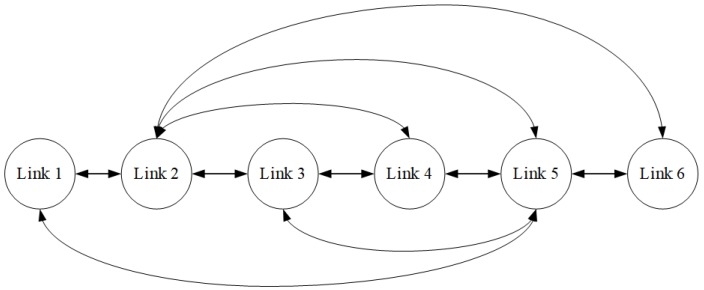
Link interference graph used in the simulations.

We simulated a network with six links where Links 1, 2, 3 were real-time links and Links 4, 5, 6 were non-real-time links. The interference set of each link C(i) is defined as follows: C(1) = {2, 5}; C(2) = {1, 3, 4, 5, 6}; C(3) = {2, 4, 5}; C(4) = {2, 3, 5}; C(5) = {1, 2, 3, 4, 6}; C(6) = {2, 5}. In order to be more intuitive, the interference graph is given by Figure 3 in which connections between links indicate interference between the links.

The probability that a packet arrives at a link in a time slot is assumed to have a Bernoulli distribution with parameter λ. We changed the arrival rate λ from 0–0.8 with an increment of 0.05 in our simulations to test performance under various traffic loads.

In this paper, the control slot is further divided into 48 control mini-slots, (which lies in the range of the backoff window size specified in IEEE 802.11 DCF [32]). The expiration time of the decision packet of the real-time link is a random integer in the interval [5, 30]. The virtual expiration time of the decision packet of the non-real-time link (that is, the decision packet is not successfully transmitted to the fusion center when the virtual remaining lifetime is zero, and the decision packet is not discarded) was set to a fixed integer 30. In the dynamic priority formula, δ was set to 0.1; $w_{1}$ took 0.4; and $w_{2}$ was 0.6. $M_{i}^{k}\left(0\right)$ and $V_{i}^{k}\left(0\right)$ were equal to zero. *K* was 10. The mapping relationship between $P_{i1}$ and $w_{i}$ can be viewed in Table 1.

The parameters for the scheduling algorithm used for comparison were set as follows:

**DMS**: W=48.
**QCSMA**: W=48, $w_{i}\left(t\right)=log\left(0.1q_{i}\left(t\right)\right)$.
**GDCSMA**: W=48, $\delta_{i}=0.9$, $\rho_{i}=0.4$. K=10.

### 6.2. Simulation Results Analysis

The scheduling set obtained by the scheduling algorithm proposed in this paper was the maximum feasible scheduling set for each time slot in the goal in Section 3, that is the set of links that interfered with each other as little as possible. The work of this paper simulated the case where the packet arrival rate λ varied from 0–0.8, and the total throughput, throughput of real-time links, throughput of non-real-time links, delay missing probability of real-time links, and value missing probability of real-time links were counted. The simulation results show that the throughput of the proposed scheduling algorithm was high, especially in the case of the higher arrival rate; that is, the busier the network is, the higher the throughput of real-time links and the lower the value missing probability of real-time links were. This paper considers the situation that the network traffic is too large, that is the data packet arrival rate was getting higher and higher in the simulation experiment. When the load in the network was excessively increased, the network was congested. When the network was congested, data loss generally occurred, and the delay increased. As throughput decreased, network performance was bound to decline. However, in order to ensure the performance of IoT data fusion, the proposed algorithm could ensure that when the network was congested, it could still transmit more real-time data packets to the fusion center within its deadline and could transmit as many valuable packets as possible to the fusion center.

**Figure 4:**
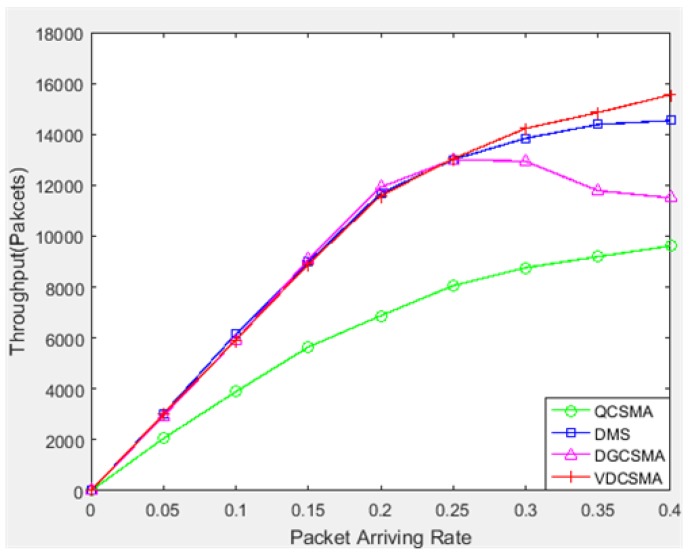
Total throughput: λ∈[0,0.4].

**Figure 5:**
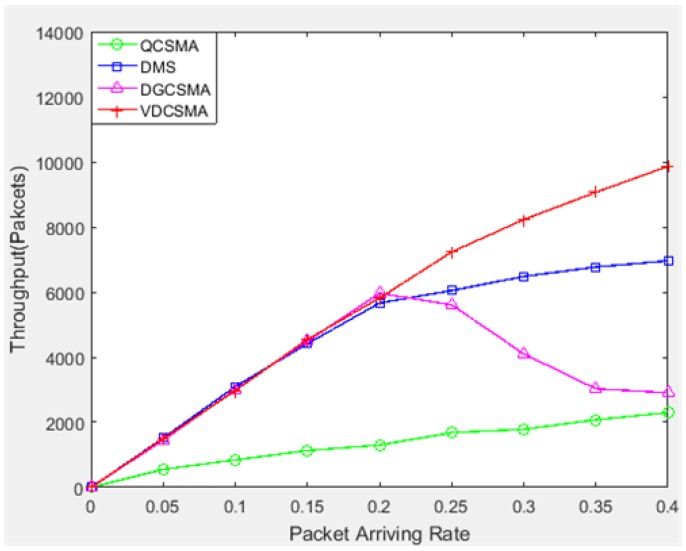
Throughput of real-time links: λ∈[0,0.4].

**Figure 6:**
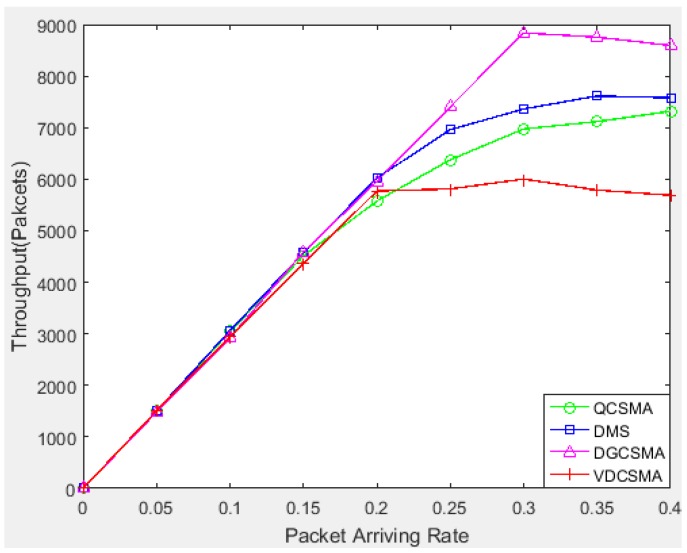
Throughput of non-real-time links: λ∈[0,0.4].

Figure 4, Figure 5 and Figure 6 show the simulation results for the throughput performance of the scheduling algorithm under relatively light traffic load (packet arrival rate λ varied from 0–0.4). Figure 4 shows the total throughput of the four scheduling algorithms. It can be seen from the figure that the total throughputs of VDCSMA, DGCSMA, and DMS were similar under light traffic, while the throughput of QCSMA was lower than the other three scheduling algorithms.

Figure 5 shows the throughput of the real-time links of each algorithm under light traffic. The throughputs of VDCSMA, DGCSMA, and DMS were very similar when the packet arrival rate was between zero and 0.25. However, QCSMA for real-time converged link packet transmission performance was poor. With a packet arrival rate greater than 0.2, the VDCSMA throughput was significantly higher than the other three algorithms with an arrival rate of 0.2–0.4. This shows that the VDCSMA proposed in this paper had good performance in guaranteeing packet transmission of the real-time link under low traffic intensity.

**Figure 7:**
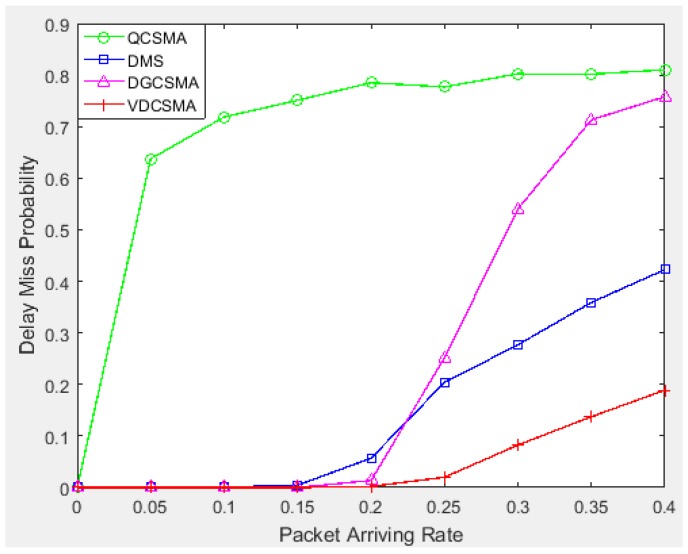
Delay missing probability of real-time links: λ∈[0,0.4].

Figure 6 indicates that the other three scheduling algorithms achieved a better throughput for non-real-time packet transmission; however, given the total amount of traffic load, high throughput for non-real-time transmission implies sacrificing the throughput of real-time packets. As can be seen from Figure 7, the delay miss probability of real-time packets of VDCSMA algorithm was much lower than those of the other three algorithms. In other words, the other three algorithms ensured non-real-time packet transmission by discarding real-time packets. However, the algorithm proposed in this paper was designed to ensure the performance of data fusion. Data fusion is sensitive to the delay of real-time data. Therefore, it is significant to reduce the throughput of non-real-time decision packets properly in order to ensure the transmission of real-time packets.

**Figure 8:**
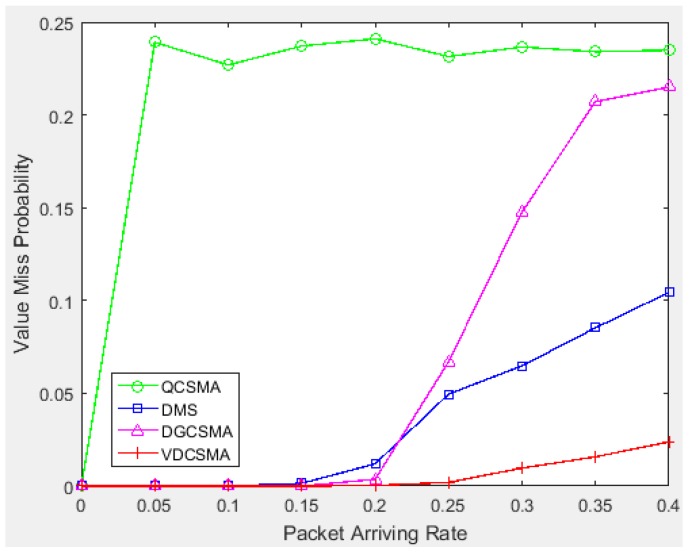
Value missing probability of real-time links: λ∈[0,0.4].

Figure 8 shows the value miss probability for real-time links. Under the condition of low traffic intensity, when the packet arrival rate was greater than 0.2, the value miss rates of DMS and DGCSMA for real-time packets began to increase. Moreover, the value miss probability of QCSMA for real-time packets was high even under light traffic. However, the value miss probability of the proposed VDCSMA was almost zero, indicating that this algorithm can guarantee the transmission of packets that are more valuable for data fusion well.

**Figure 9:**
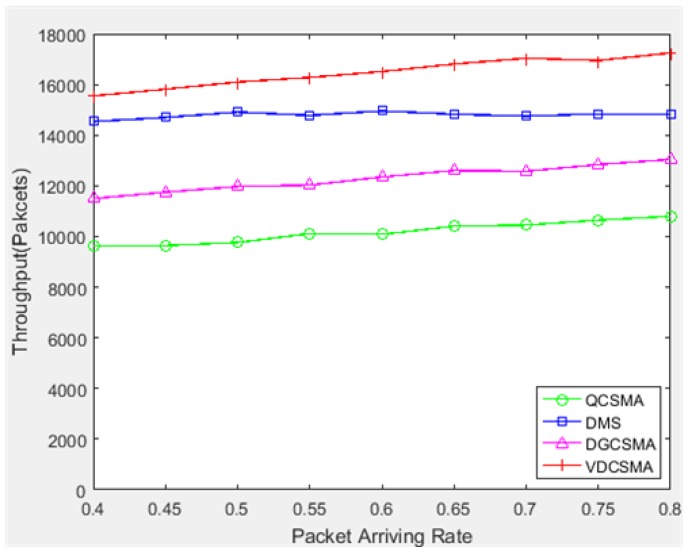
Total throughput: λ∈[0.4,0.8].

**Figure 10:**
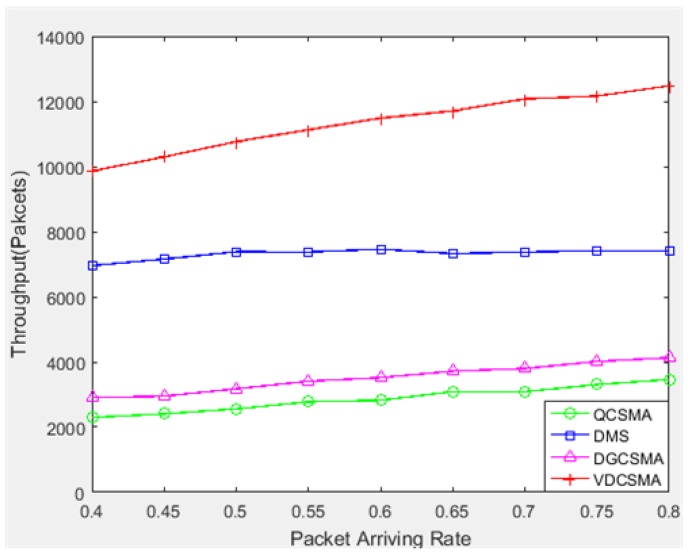
Throughput of real-time links: λ∈[0.4,0.8].

**Figure 11:**
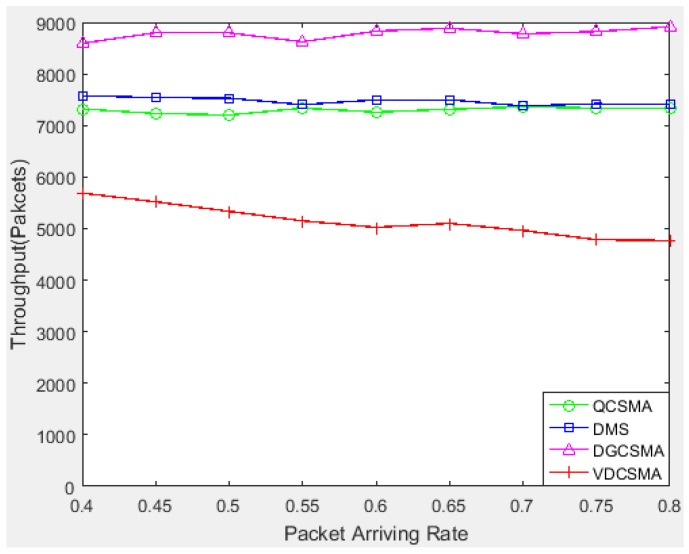
Throughput of non-real-time links: λ∈[0.4,0.8].

**Figure 12:**
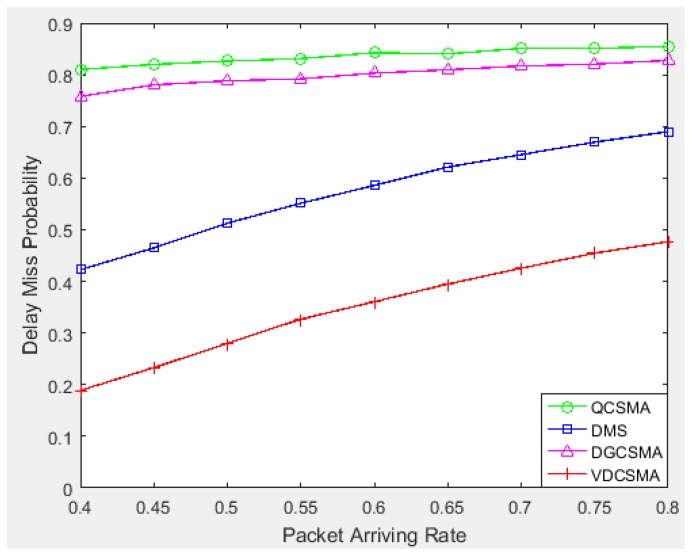
Delay missing probability of real-time links: λ∈[0.4,0.8].

**Figure 13:**
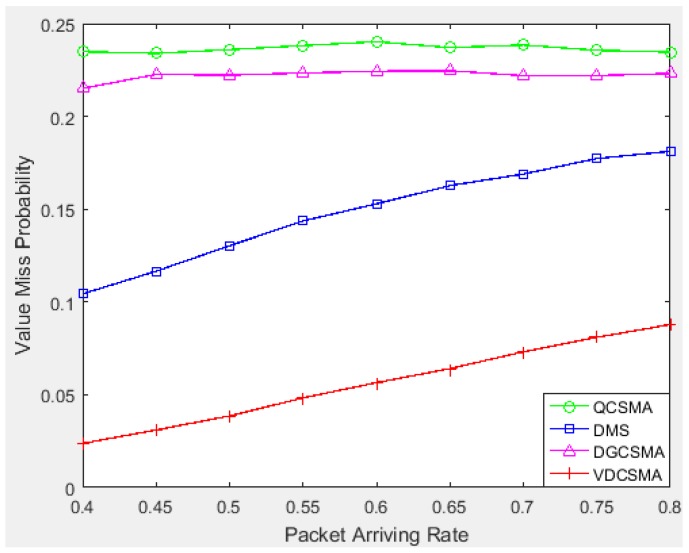
Value missing probability of real-time links: λ∈[0.4,0.8].

Figure 9, Figure 10, Figure 11, Figure 12 and Figure 13 give the performance comparison results under relatively heavy traffic load (arrival rate λ varied from 0.4–0.8). Figure 9 gives the total throughput of the system, which shows that the throughput of all four algorithms increased with the packet arrival rate. The total throughput of VDCSMA was higher than the other three algorithms.

Figure 10 and Figure 11 respectively give the throughput performance of real-time and non-real-time links. Similar to the light traffic case, VDCSMA achieved the best throughput for real-time packet transmission among these algorithms at the cost of the non-real-time packet throughput.

Figure 12 gives the delay missing probabilities achieved by the tested algorithms for real-time links. We can see from this figure that the delay missing probabilities for all four algorithms inevitably increased when traffic load became heavier, but VDCSMA maintained the lowest delay missing rate, which shows that VDCSMA could better ensure the transmission of real-time packets.

When it is inevitable to discard some real-time packets due to missing the delay deadline under heavy traffic, it becomes more important to ensure transmitting packets that are more valuable to data fusion. Figure 13 gives the value missing rates of the four tested algorithms, from which we can see that VDCSMA achieved the lowest value missing rate, thus ensuring the transmission of more valuable packets as much as possible.

## 7. Conclusions

In this paper, we proposed a distributed scheduling scheme VD-CSMA for enhancing the throughput and delay performance of data transmission in a wireless sensor network for supporting data fusion in an IoT environment. The proposed scheduling algorithm employs a dynamic priority assignment scheme, which determines the priority level for each packet based on multiple factors including the value of the packet to data fusion, as well as the urgency of the packet with respect to its transmission deadline. The scheme assigns higher priority levels to packets that contain more valuable data for data fusion and closer to their deadlines; thus allowing such packets to obtain better chances for transmission in a distributed scheduling system. Experimental results obtained from extensive simulations verified that VD-CSMA achieves good performance under both light and heavy traffic loads. The proposed algorithm has low complexity and is suitable for distributed implementation.

## Figures and Tables

**Table 1:** Mapping relationship between $P_{i1}$ and $w_{i}$.

$P_{i1}$	$w_{i}$
[1.25, ∞)	[0, 7]
[1, 1.25)	[8, 15]
[0.75, 1)	[16, 23]
[0.5, 0.75)	[24, 31]
[0.25, 0.5)	[32, 39]
[0, 0.25)	[40, 47]
